# The Growth and Scope of Voluntary Hospitals in London

**Published:** 1897-08-28

**Authors:** 


### The Institutional Workshop.

### THE GROWTH AND SCOPE OF VOLUNTARY HOSPITALS IN LONDON.

From a Correspondent.

### VI.—RISE OF THE MODERN DISPENSARIES.

The gratuitous medical work of the College of Physicians at the three dispensaries in Warwick Lane, St. Martin's Lane, and Gracechurch Street appears, as far as can be judged from the scanty records of it which have survived, to have depended on the exertions of a few charitable individuals. Free advice on application, medicine at cost price, and more rarely attendance at the homes of those unable to move, were certainly provided to the poor by the dispensarians for several years at the beginning of the century. But owing to the bitter persecution to which they were exposed, not only from the apothecaries, but also from members of their own profession, the scheme which had been intended to comprise the relief of all the sick poor of London was soon limited to the private exertions of a few persons, and was carried on with a degree of secrecy very unusual in undertakings of this description. An anonymous writer, who undertook the defence of these charitable doctors in 1708, complains that "the citizens are unhappily ignorant of the care the physicians and surgeons give to the servants and indigent near them." It would seem that as the original dispensarians died out they found no successors, and the dispensaries gradually fell into disuse. No mention is made of them in the preambles to any of the new medical charities of the century, and the neglected state of the sick poor is attested by many contemporary writers.

It was the promoters of the lying-in charities who first devised the expedient of relieving in their own homes patients who were unable or unwilling to become inmates of a hospital. But it was some years before this new method of practice was applied to other than lying-in patients. The need was very grave. There were many cases which hospitals were prevented by pressure of space from taking, such as asthma, consumption, chronic sores, and infectious fevers; there were then as now far more applications at the general hospitals than could be entertained, and there were again cases in which removal would have been fatal. In view of so much unrelieved wretchedness a scheme was devised by Dr. Hulme, ably assisted by Dr. Lettsom, for ministering to the poor in their own homes, and the result was the establishment, in 1771, of the General Dispensary. The wise constitution on which the two physicians framed this dispensary made it a model for nearly all the others subsequently founded in this and other countries. As in the case of voluntary hospitals, the government was vested in the hands of the subscribers. A yearly subscription of one guinea or a donation of ten guineas constituted the subscriber a governor; and the management was vested in the hands of 16 governors, acting with the president, vice-president, and treasurer. A standing medical committee was framed of "all such governors as practice physic, surgery, or pharmacy," and the appointment of the medical officers rested with this medical board. Only members of the Royal College of Physicians, the Royal College of Surgeons, and the Apothecaries' Company were eligible for these offices; a good deal of prestige was conferred by the posts as well as unrivalled opportunities for study and observation, and the competition for them became before long very keen. From a pamphlet of Dr. Lettsom's describing the progress of the new scheme, it appears that "a genteel emolument of £100 was bestowed on the physicians for their labours during the year." Some alarm appears to have been caused in the profession from the dread of losing their patients; and that this misgiving was not wholly without foundation is clear from the admission that "probably individuals in easy circumstances may have assumed the appearance of necessity, and thereby partaken of the charity which was designed only for the indigent." Evidently a tendency to abuse medical charities has always been a marked trait in the Londoner. But at the General Dispensary they were forewarned, and well prepared to resist the evil; for we learn that "a committee of governors attend daily to prevent impositions and rectify abuse." Another class of practitioners had good reason to lament the establishment of the new institution. Dr. Lettsom boasts "the dispensary has proved a noble check to impostures in physic, and nearly extirpated medical quacks out of the precincts of the city."

Such patients as could attend the dispensary, which was situated in Aldersgate Street, were required to do so; the remainder were visited in their own homes. Dr. Lettsom plunged into the work of visitation with a vehemence and enthusiasm which infected his colleagues, and completely disarmed the dull suspiciousness of the poor. The worst scourge of the more closely-inhabited quarters at that time was a kind of putrid fever, engendered by semi-starvation and foul air. The traditional treatment of this complaint, under which thousands had perished, was close confinement to bed under heavy wrappings, accompanied by a system of sweating, purging, and bleeding. Dr. Lettsom got all his patients out of bed and out of doors with the fever still on them. If they were too feeble to support themselves, he opened doors and windows, and set them lightly clad in a steady draught of cool air. For drugs he relied on Peruvian bark, and copious draughts of good London porter. The effects were miraculous, and if the fresh air was never an exactly palatable prescription the porter redeemed the day.

The benefits of the visiting part of the charity were confined to the City and its liberties, but no limit was placed on the area from which out-patients were drawn. The charity was for some years after its opening purely medical.

One of the pleas urged for the establishment of the new dispensary was the high rate of infant mortality, and the fact that no provision existed in general hospitals for the cure of young children. No vital statistics of any scientific value are available for this period, or till much later. But it was estimated by those who had studied the matter most carefully that nearly half the number of children born in London died under two years of age. This slaughter of the innocents appealed forcibly to the charitable, and shortly before the founding of the General Dispensary an attempt was made by a private individual to aid the little sufferers of the metropolis. To Dr. George Armstrong belongs the honour of opening the first institution for giving free medicine and advice to the poor in his Dispensary for the Relief of the Infant Poor, founded in 1769. Although subscriptions were invited, Dr. Armstrong bore for a considerable time nearly the whole expense and labour of the undertaking on his own shoulders. The dispensary was situated in Red Lion Square, and attendance was given on three days in the week for free patients, and on Saturdays for paying patients. The number of patients relieved rose from 696 in the first year to 1,296 in the second, and at the end of the third year the numbers were so considerable (sometimes amounting to as many as 80 in a day) that an effort was made to place the enterprise on a more regular basis on the model of the General Dispensary. It does not appear, however, that the scheme was one of very long continuance, and it is noteworthy principally as the first attempt to deal systematically with the diseases of children.

